# Medical Diagnostic Tests: A Review of Test Anatomy, Phases, and Statistical Treatment of Data

**DOI:** 10.1155/2019/1891569

**Published:** 2019-05-28

**Authors:** Sorana D. Bolboacă

**Affiliations:** Department of Medical Informatics and Biostatistics, Iuliu Hațieganu University of Medicine and Pharmacy Cluj-Napoca, Louis Pasteur Str., No. 6, 400349 Cluj-Napoca, Romania

## Abstract

Diagnostic tests are approaches used in clinical practice to identify with high accuracy the disease of a particular patient and thus to provide early and proper treatment. Reporting high-quality results of diagnostic tests, for both basic and advanced methods, is solely the responsibility of the authors. Despite the existence of recommendation and standards regarding the content or format of statistical aspects, the quality of what and how the statistic is reported when a diagnostic test is assessed varied from excellent to very poor. This article briefly reviews the steps in the evaluation of a diagnostic test from the anatomy, to the role in clinical practice, and to the statistical methods used to show their performances. The statistical approaches are linked with the phase, clinical question, and objective and are accompanied by examples. More details are provided for phase I and II studies while the statistical treatment of phase III and IV is just briefly presented. Several free online resources useful in the calculation of some statistics are also given.

## 1. Introduction

An accurate and timely diagnostic with the smallest probability of misdiagnosis, missed diagnosis, or delayed diagnosis is crucial in the management of any disease [1, 2]. The diagnostic is an evolving process since both disease (the likelihood and the severity of the disease) and diagnostic approaches evolve [3]. In clinical practice, it is essential to correctly identify the diagnostic test that is useful to a specific patient with a specific condition [4–6]. The over- or underdiagnostic closely reflects on unnecessary or no treatment and harms both the subjects and the health-care systems [3].

Statistical methods used to assess a sign or a symptom in medicine depend on the phase of the study and are directly related to the research question and the design of the experiment (Table 1) [7].

A significant effort was made to develop the standards in reporting clinical studies, both for primary (e.g., case-control studies, cohort studies, and clinical trials) and secondary (e.g., systematic review and meta-analysis) research. The effort led to the publication of four hundred twelve guidelines available on the EQUATOR Network on April 20, 2019 [8]. Each guideline is accompanied by a short checklist describing the information needed to be present in each section and also include some requirements on the presentation of statistical results (information about what, e.g., mean (SD) where SD is the standard deviation, and how to report, e.g., the number of decimals). These guidelines are also used as support in the critical evaluation of an article in evidence-based clinical practice. However, insufficient attention has been granted to the minimum set of items or methods and their quality in reporting the results. Different designs of experiments received more attention, and several statistical guidelines, especially for clinical trials, were developed to standardize the content of the statistical analysis plan [9], for phase III clinical trials in myeloid leukemia [10], pharmaceutical industry-sponsored clinical trials [11], subgroup analysis [12], or graphics and statistics for cardiology [13]. The SAMPL Guidelines provide general principles for reporting statistical methods and results [14]. SAMPL recommends to provide numbers with the appropriate degree of precision, the sample size, numerator and denominator for percentages, and mean (SD) (where SD = standard deviation) for data approximately normally distributed; otherwise medians and interpercentile ranges, verification of the assumption of statistical tests, name of the test and the tailed (one- or two-tailed), significance level (*α*), *P* values even statistically significant or not, adjustment(s) (if any) for multivariate analysis, statistical package used in the analysis, missing data, regression equation with regression coefficients for each explanatory variable, associated confidence intervals and *P* values, and models' goodness of fit (coefficient of determination) [14]. In regard to diagnostic tests, standards are available for reporting accuracy (QUADAS [15], QUADAS-2 [16], STARD [17, 18], and STARD 2015 [19]), diagnostic predictive models (TRIPOD [20]), systematic reviews and meta-analysis (AMSTAR [21] and AMSTAR 2 [22]), and recommendations and guidelines (AGREE [23], AGREE II [24], and RIGHT [25]). The requirements highlight what and how to report (by examples), with an emphasis on the design of experiment which is mandatory to assure the validity and reliability of the reported results. Several studies have been conducted to evaluate if the available standards in reporting results are followed. The number of articles that adequately report the accuracy is reported from low [26–28] to satisfactory [29], but not excellent, still leaving much room for improvements [30–32].

The diagnostic tests are frequently reported in the scientific literature, and the clinicians must know how a good report looks like to apply just the higher-quality information collected from the scientific literature to decision related to a particular patient. This review aimed to present the most frequent statistical methods used in the evaluation of a diagnostic test by linking the statistical treatment of data with the phase of the evaluation and clinical questions.

## 2. Anatomy of a Diagnostic Test

A diagnostic test could be used in clinical settings for confirmation/exclusion, triage, monitoring, prognosis, or screening (Table 2) [19, 38]. Table 2 presents the role of a diagnostic test, its aim, and a real-life example.

Different statistical methods are used to support the results of a diagnostic test according to the question, phase, and study design. The statistical analysis depends on the test outcome type.  Table 3 presents the most common types of diagnostic test outcome and provides some examples.

The result of an excellent diagnostic test must be accurate (the measured value is as closest as possible by the true value) and precise (repeatability and reproducibility of the measurement) [65]. An accurate and precise measurement is the primary characteristic of a valid diagnostic test.

The reference range or reference interval and ranges of normal values determined in healthy persons are also essential to classify a measurement as a positive or negative result and generally refer to continuous measurements. Under the assumption of a normal distribution, the reference value of a diagnostic measurement had a lower reference limit/lower limit of normal (LRL) and an upper reference limit/upper limit of normal (URL) [66–71]. Frequently, the reference interval takes the central 95% of a reference population, but exceptions from this rule are observed (e.g., cTn-cardiac troponins [72] and glucose levels [73] with <5% deviation from reference intervals) [74, 75]. The reference ranges could be different among laboratories [76, 77], genders and/or ages [78], populations [79] (with variations inclusive within the same population [80, 81]), and to physiological conditions (e.g., pregnancy [82], time of sample collection, or posture). Within-subject biological variation is smaller than the between-subject variation, so reference change values could better reflect the changes in measurements for an individual as compared to reference ranges [83]. Furthermore, a call for establishing the clinical decision limits (CDLs) with the involvement of laboratory professionals had also been emphasized [84].

The *Z*-score (standardized value, standardized score, or *Z*-value, *Z*-score = (measurement − *μ*)/*σ*)) is a dimensionless metric used to evaluate how many standard deviations (*σ*) a measurement is far from the population mean (*μ*) [85]. A *Z*-score of 3 refers to 3 standard deviations that would mean that more than 99% of the population was covered by the *Z*-score [86]. The *Z*-score is properly used under the assumption of normal distribution and when the parameters of the population are known [87]. It has the advantage that allows comparing different methods of measurements [87]. The *Z*-scores are used on measurements on pediatric population [88, 89] or fetuses [90], but not exclusively (e.g., bone density tests [91]).

## 3. Diagnostic Tests and Statistical Methods

The usefulness of a diagnostic test is directly related with its reproducibility (the result is the same when two different medical staff apply the test), accuracy (the same result is obtained if the diagnostic test is used more than once), feasibility (the diagnostic method is accessible and affordable), and the effect of the diagnostic test result on the clinical decision [92]. Specific statistical methods are used to sustain the utility of a diagnostic test, and several examples linking the phase of a diagnostic test with clinical question, design, and statistical analysis methods are provided in Table 4 [101].

### 3.1. Descriptive Metrics

A cohort cross-sectional study is frequently used to establish the normal range of values. Whenever data follow the normal distribution (normality tests such as Shapiro–Wilk [102] or Kolmogorov–Smirnov test [103, 104] provide valid results whenever the sample sizes exceed 29), the mean and standard deviations are reported [105], and the comparison between groups is tested with parametric tests such as Student's *t*-test (2 groups) or ANOVA test (more than 2 groups). Median and quartiles (*Q*1 − *Q*3) are expected to be reported, and the comparison is made with nonparametric tests if experimental data did not follow the normal distribution or the sample size is less than 30 [105]. The continuous data are reported with one or two decimals (sufficient to assure the accuracy of the result), while the *P* values are reported with four decimals even if the significance threshold was or not reached [106].

The norms and good practice are not always seen in the scientific literature while the studies are frequently more complex (e.g., investigation of changes in the values of biomarkers with age or comparison of healthy subjects with subjects with a specific disease). One example is given by Koch and Singer [107], which aimed to determine the range of normal values of the plasma B-type natriuretic peptide (BNP) from infancy to adolescence. One hundred ninety-five healthy subjects, infants, children, and adolescents were evaluated. Even that the values of BNP varied considerably, the results were improper reported as mean (standard deviation) on the investigated subgroups, but correctly compared subgroups using nonparametric tests [107, 108]. Taheri et al. compared the serum levels of hepcidin (a low molecular weight protein role in the iron metabolism) and prohepcidin in hemodialysis patients (44 patients) and healthy subjects (44 subjects) [93]. Taheri et al. reported the values of hepcidin and prohepcidin as a mean and standard deviation, suggesting the normal distribution of data, and compared using nonparametric tests, inducing the absence of normal distribution of experimental data [93]. Furthermore, they correlated these two biomarkers while no reason exists for this analysis since one is derived from the other [93].

Zhang et al. [94] determined the reference values for plasma pro-gastrin-releasing peptide (ProGrP) levels in healthy Han Chinese adults. They tested the distribution of ProGrP, identified that is not normally distributed, and correctly reported the medians, ranges, and 2.5^th^, 5^th^, 50^th^, 95^th^, and 97.5^th^ percentiles on two subgroups by ages. Spearman's correlation coefficient was correctly used to test the relation between ProGrP and age, but the symbol of this correlation coefficient was *r* (symbol attributed to Pearson's correlation coefficient) instead of *ρ*. The differences in the ProGrP among groups were accurately tested with the Mann–Whitney test (two groups) and the Kruskal–Wallis test (more than two groups). The authors reported the age-dependent reference interval on this specific population without significant differences between genders [94].

The influence of the toner particles on seven biomarkers (serum C-reactive protein (CRP), IgE, interleukin (IL-4, IL-6, and IL-8), serum interferon-*γ* (IFN-*γ*), and urine 8-hydroxy-2′-deoxyguanosine (8OHdG)) was investigated by Murase et al. [109]. They conducted a prospective cohort study (toner exposed and unexposed) with a five-year follow-up and measured annually the biomarkers. The reference values of the studied biomarkers were correctly reported as median and 27^th^–75^th^ percentiles as well as the 2.5^th^–97.5^th^ percentiles (as recommended by the Clinical and Laboratory Standards Institute [108]).

### 3.2. Variability Analysis

Two different approaches are used whenever variability of quantitative data is tested in phase I studies, both reflecting the repeated measurements (the same or different device or examiner), namely, variation analysis (coefficient of variation, CV) or the agreement analysis (agreement coefficients).

#### 3.2.1. Variation Analysis

Coefficient of variation (CV), also known as relative standard deviation (RSD), is a standardized measure of dispersion used to express the precision (intra-assay (the same sample assayed in duplicate) CV < 10% is considered acceptable; interassay (comparison of results across assay runs) CV < 15% is deemed to be acceptable) of an assay [110–112]. The coefficient of variation was introduced by Karl Pearson in 1896 [113] and could also be used to test the reliability of a method (the smaller the CV values, the higher the reliability is) [114], to compare methods (the smallest CV belongs to the better method) or variables expressed with different units [115]. The CV is defined as the ratio of the standard deviation to the mean expressed as percentage [116] and is correctly calculated on quantitative data measured on the ratio scale [117]. The coefficient of quartile variation/dispersion (CQV/CQD) was introduced as a preferred measure of dispersion when data did not follow the normal distribution [118] and was defined based on the third and first quartile as (*Q*3 – *Q*1)/(*Q*3 + *Q*1)^*∗*^100 [119]. In a survey analysis, the CQV is used as a measure of convergence in experts' opinions [120].

The confidence interval associated with CV is expected to be reported for providing the readers with sufficient information for a correct interpretation of the reported results, and several online implementations are available (Table 5).

The inference on CVs can be made using specific statistical tests according to the distribution of data. For normal distributions, tests are available to compare two [121] or more than two CVs (Feltz and Miller test [122] or Krishnamoorthy and Lee test [123], the last one also implemented in R [124]).

Reporting the CVs with associated 95% confidence intervals allows a proper interpretation of its point estimator value (CV). Schafer et al. [125] investigated laboratory reproducibility of urine N-telopeptide (NTX) and serum bone-specific alkaline phosphatase (BAP) measurements with six labs over eight months and correctly reported the CVs with associated 95% confidence intervals. Furthermore, they also compared the CVs between two assays and between labs and highlighted the need for improvements in the analytical precision of both NTX and BAP biomarkers [125]. They concluded with the importance of the availability of laboratory performance reports to clinicians and institutions along with the need for proficiency testing and standardized guidelines to improve market reproducibility [125].

However, good practice in reporting CVs is not always observed. Inter- and intra-assay CVs within laboratories reported by Calvi et al. [126] on measurements of cortisol in saliva are reported as point estimators, and neither confidence intervals nor statistical test is provided. Reed et al. [127] reported the variability of measurements (thirty-three laboratories with fifteen repeated measurements on each lab) of human serum antibodies against *Bordetella pertussis* antigens by ELISA method using just the CVs (no associated 95% confidence intervals) in relation with the expected fraction of pairs of those measurements that differ by at least a given factor (*k*).

#### 3.2.2. Agreement Analysis

Percentage agreement (*p*_*o*_), the number of agreements divided into the number of cases, is the easiest agreement coefficient that could be calculated but may be misleading. Several agreement coefficients that adjust the proportional agreement by the agreement expected by chance were introduced:

- Nominal or ordinal scale: Cohen's kappa coefficient (nominal scale, inclusive dichotomial such as positive/negative test result), symbol *κ* [128], and its derivatives (Fleiss' generalized kappa [129], Conger's generalized kappa [130], and weighted kappa (ordinal scale test result)) [131]
- Numerical scale: intraclass (Pearson's correlation coefficient (*r*)) [132] and interclass correlation coefficient (ICC) [133] (Lin's concordance correlation coefficient (*ρ*_*c*_) [134, 135] and Bland and Altman diagram (B&A plot [136, 137]))

The Cohen's kappa coefficient has three assumptions: (i) the units are independent, (ii) the categories on the nominal scale are independent and mutually exclusive, and (iii) the readers/raters are independent [128]. Cohen's kappa coefficient takes a value between −1 (perfect disagreement) and 1 (complete agreement). The empirical rules used to interpret the Cohen's kappa coefficient [138] are as follows: no agreement for *κ* ≤ 0.20, minimal agreement for 0.21 < *κ* ≤ 0.39, week agreement for 0.40 ≤ *κ* ≤ 0.59, moderate agreement for 0.60 ≤ *κ* ≤ 0.79, strong agreement for 0.80 ≤ *κ* ≤ 0.90, and almost perfect agreement for *κ* > 0.90. The minimum acceptable interrater agreement for clinical laboratory measurements is 0.80. The 95% CI must accompany the value of *κ* for a proper interpretation, and the empirical interpretation rules must apply to the lower bound of the confidence interval.

The significance of *κ* could also be calculated, but in many cases, it is implemented to test if the value of *κ* is significantly different by zero (*H*_*0*_*(null hypothesis): κ* *=* *0*). The clinical significance value is 0.80, and a test using the null hypothesis as *H*_*0*_*: κ* *=* *0.79* vs. *H*_*1*_*(one-sided alternative hypothesis): κ* *>* *0.79* should be applied.

Weighted kappa is used to discriminate between different readings on ordinal diagnostic test results (different grade of disagreement exists between *good* and *excellent* compared to *poor* and *excellent*). Different weights reflecting the importance of agreement and the weights (linear, proportional to the number of categories apart or quadratic, proportional to the square of the number of classes apart) must be established by the researcher [131].

Intra- and interclass correlation coefficients (ICCs) are used as a measure of reliability of measurements and had their utility in the evaluation of a diagnostic test. Interrater reliability (defined as two or more raters who measure the same group of individuals), test-retest reliability (defined as the variation in measurements by the same instrument on the same subject by the same conditions), and intrarater reliability (defined as variation of data measured by one rater across two or more trials) are common used [139]. McGraw and Wong [140] defined in 1996 the ten forms of ICC based on the *model* (1-way random effects, 2-way random effects, or 2-way fixed effects), the *number of rates/measurements* (single rater/measurement or the mean of *k* raters/measurements), and *hypothesis* (consistency or absolute agreement). McGraw and Wong also discuss how to correctly select the correct ICC and recommend to report the ICC values along with their 95% CI [140].

Lin's concordance correlation coefficient (*ρ*_*c*_) measures the concordance between two observations, one measurement as the *gold standard*. The ranges of values of Lin's concordance correlation coefficient are the same as for Cohen's kappa coefficient. The interpretation of *ρ*_*c*_ takes into account the scale of measurements, with more strictness for continuous measurements (Table 6) [141, 142]. For intra- and interobserver agreement, Martins and Nastri [142] introduced the metric called limits of agreement (LoA) and proposed a cutoff < 5% for very good reliability/agreement.

Reporting the ICC and/or CCC along with associated 95% confidence intervals is good practice for agreement analysis. The results are reported in both primary (such as reliability analysis of the Microbleed Anatomical Rating Scale in the evaluation of microbleeds [143], automatic analysis of relaxation parameters of the upper esophageal sphincter [144], and the use of signal intensity weighted centroid in magnetic resonance images of patients with discs degeneration [145]) and secondary research studies (systematic review and/or meta-analysis: evaluation of the functional movement screen [146], evaluation of the Manchester triage scale on an emergency department [147], reliability of the specific physical examination tests for the diagnosis of shoulder pathologies [148], etc.).

Altman and Bland criticized the used of correlation (this is a measure of association, and it is not correct to infer that the two methods can be used interchangeably), linear regression analysis (the method has several assumptions that need to be checked before application, and the assessment of residuals is mandatory for a proper interpretation), and the differences between means as comparison methods aimed to measure the same quantity [136, 149, 150]. They proposed a graphical method called the B&A plot to analyze the agreement between two quantitative measurements by studying the mean difference and constructing limits of agreement [136, 137]. Whenever a *gold standard* method exists, the difference between the two methods is plotted against the reference values [151]. Besides the fact that the B&A plot provides the limits of agreements, no information regarding the acceptability of the boundaries is supplied, and the acceptable limits must be a priori defined based on clinical significance [150]. The B&A plot is informally interpreted in terms of bias (*How big the average discrepancy between the investigated methods is? Is the difference large enough to be clinically relevant?*), equivalence (*How wide are the limits of agreement?*, limits wider than those defined clinically indicate ambiguous results while narrow and small bias suggests that the two methods are equivalent), and trend and variability (*Are the dots homogenous*?).

Implementation of the 95% confidence intervals associated to ICC, CCC, or kappa statistics and the test of significance are implemented in commercial or free access statistical programs (such as SPSS, MedCalc, SAS, STATA, R, and PASS-NCSS) or could be found freely available online (e.g. vassarstats-©Richard Lowry 2001–2018, http://vassarstats.net/kappa.html; KappaCalculator ©Statistics Solutions 2018, http://www.statisticssolutions.com/KappaCalculator.html; and KappaAcc-Bakeman's Programs, http://bakeman.gsucreate.org/kappaacc/; all accessed August 27, 2018)).

### 3.3. Accuracy Analysis

The accuracy of a diagnostic test is related to the extent that the test gives the right answer, and the evaluations are done relative to the best available test (also known as *gold standard* test or *reference* test and hypothetical ideal test with sensitivity (Se) = 100% and specificity (Sp) = 100%) able to reveal the right answer. Microscopic examinations are considered the *gold standard* in the diagnosis process but could not be applied to any disease (e.g., stable coronary artery disease [152], rheumatologic diseases [153], psychiatric disorders [154], and rare diseases with not yet fully developed histological assessment [155]).

The factors that could affect the accuracy of the diagnostic test can be summarized as follows [156, 157]: sampling bias, imperfect *gold standard* test, artefactual variability (e.g., changes in prevalence due to inappropriate design) or clinical variability (e.g., patient spectrum and “gold-standard” threshold), subgroups differences, or reader expectations.

Several metrics calculated based on the 2 × 2 contingency table are frequently used to assess the accuracy of a diagnostic test. A *gold standard* or *reference* test is used to classify the subject either in the group with the disease or in the group without the disease of interest. Whatever the type of data for the diagnostic test is, a 2 × 2 contingency table can be created and used to compute the accuracy metrics. The generic structure of a 2 × 2 contingency table is presented in Table 7, and if the diagnostic test is with high accuracy, a significant association with the reference test is observed (significant Chi-square test or equivalent (for details, see [158])).

Several standard indicators and three additional metrics useful in the assessment of the accuracy of a diagnostic test are briefly presented in Tables 8 and 9.

The reflection of a positive or negative diagnosis on the probability that a patient has/not a particular disease could be investigated using Fagan's diagram [165]. The Fagan's nomogram is frequently referring in the context of evidence-based medicine, reflecting the decision-making for a particular patient [166]. The Bayes' theorem nomogram was published in 2011, the method incorporating in the prediction of the posttest probability the following metrics: pretest probability, pretest odds (for and against), PLR or NLR, posttest odds (for and against), and posttest probability [167]. The latest form of Fagan's nomogram, called two-step Fagan's nomogram, considered pretest probability, Se (Se of test for PLR), LRs, and Sp (Sp of test for NLR), in predicting the posttest probability [166].

The receiver operating characteristic (ROC) analysis is conducted to investigate the accuracy of a diagnostic test when the outcome is quantitative or ordinal with at least five classes [168, 169]. ROC analysis evaluates the ability of a diagnostic test to discriminate positive from negative cases. Several metrics are reported related to the ROC analysis in the evaluation of a diagnostic test, and the most frequently used metrics are described in Table 10 [170, 171]. The closest the left-upper corner of the graph, the better the test. Different metrics are used to choose the cutoff for the optimum Se and Sp, such as Youden's index (*J*, maximum), *d*^2^ ((1 − Se)^2^ + (1 − Sp)^2^, minimum), the weighted number needed to misdiagnose (maximum, considered the pretest probability and the cost of a misdiagnosis) [172], and Euclidean index [173]. The metrics used to identify the best cutoff value are a matter of methodology and are not expected to be reported as a result (reporting a *J* index of 0.670 for discrimination in small invasive lobular carcinoma [174] is not informative because the same *J* could be obtained for different values of Se and Sp: 0.97/0.77, 0.7/0.97, 0.83/0.84, etc.). Youden's index has been reported as the best metric in choosing the cutoff value [173] but is not able to differentiate between differences in sensitivity and specificity [175]. Furthermore, Youden's index can be used as an indicator of quality when reported with associated 95% confidence intervals, and a poor quality being associated with the presence of 0.5 is the confidence interval [175].

### 3.4. Performances of a Diagnostic Test by Examples

The body mass index (BMI) was identified as a predictor marker of breast cancer risk on Iranian population [176], with an AUC 0.79 (95% CI: 0.74 to 0.84).

**Figure 1:**
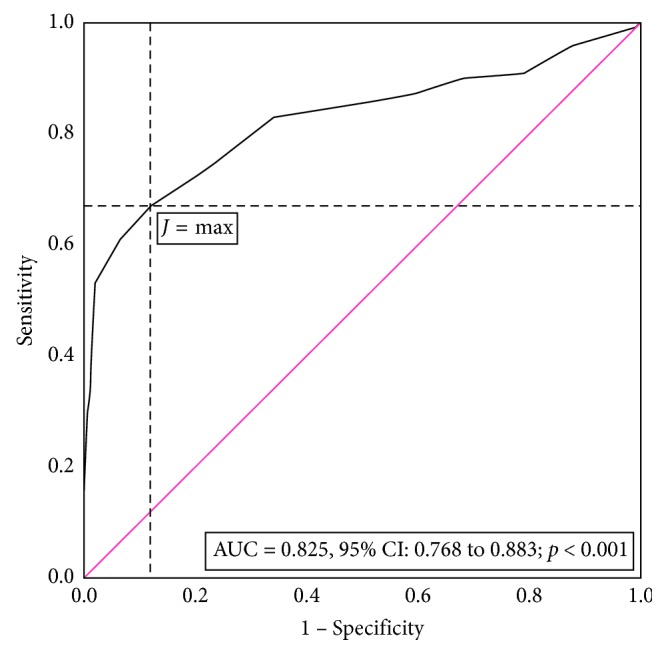
Summary receiver operating characteristic (ROC) curve for BMI as an anthropometric marker to distinguish benign from malign breast tumors. The red line shows an equal proportion of correctly classified breast cancer sample and incorrectly classifies samples without breast cancer (random classification). The *J* max (max (Se + Sp − 1)) corresponds to a Se = 0.67 and a Sp = 0.88 for a cutoff > 29.5 kg/m^2^ (BMI) for the breast cancer sample.

A simulation dataset was used to illustrate how the performances of a diagnostic test could be evaluated, evaluating the BMI as a marker for breast cancer. The simulation was done with respect to the normal distribution for 100 cases with malign breast tumor and 200 cases with benign breast tumors with BMI mean difference of 5.7 kg/m^2^ (Student's *t*-test assuming unequal variance: *t*-stat = 9.98, *p* < 0.001). The body mass index (BMI) expressed in kg/m^2^ varied from 20 to 44 kg/m^2^, and the ROC curve with associated AUC is presented in Figure 1.

The ROC curve graphically represents the pairs of Se and (1 − Sp) for different cutoff values. The AUC of 0.825 proved significantly different by 0.5 (*p* < 0.001), and the point estimator indicates a good accuracy, but if the evaluation is done based on the interpretation of the 95% lower bound, we found the BMI as a worthless test for breast cancer. The *J* had its maximum value at a cutoff equal to 29.5 kg/m^2^ and corresponded to a Se of 0.67, a Sp of 0.88, and an AI of 0.81. The PLR of 5.58 indicates that the BMI is strong diagnostic evidence, but this classification is not supported by the value of NLR which exceed the value of 0.2 (Table 10). A BMI >29.5 kg/m^2^*usually occurs* in those with breast cancer while a BMI ≤ 29.5 kg/m^2^*often occurs* in those without breast cancer. At a cutoff of 29.5 kg/m^2^, the marker is *very poor* for finding those *with breast cancer* but is good for screening.

The performance metrics varied according to the cutoff values (Table 11). A cutoff with a low value is chosen whenever the aim is to minimize the number of false negatives, assuring a Se of 1 (19.5 kg/m^2^, TP = 100, Table 10). If a test able to correctly classify the true negatives is desired, the value of the cutoff must be high (38.5 kg/m^2^, TN = 200, Table 11) assuring a Sp of 1.

The analysis of the performance metrics for our simulation dataset showed that the maximum CUI+ and CUI− values are obtained for the cutoff value identified by the *J* index, supporting the usefulness of the BMI for screening not for case finding.

The accuracy analysis is reported frequently in the scientific literature both in primary and secondary studies. Different actors such as the authors, reviewers, and editors could contribute to the quality of the statistics reported. The evaluation of plasma chitotriosidase as a biomarker in critical limb ischemia reported the AUC with associated 95% confidence intervals, cutoff values [59], but no information on patient-centered metrics or utility indications are provided. Similar parameters as reported by Ciocan et al. [59] have also been reported in the evaluation of sonoelastographic scores in the differentiation of benign by malign cervical lymph nodes [45]. Lei et al. conducted a secondary study to evaluate the accuracy of the digital breast tomosynthesis versus digital mammography to discriminate between malign and benign breast lesions and correctly reported Se, Sp, PLR, NLR, and DOR for both the studies included in the analysis and the pooled value [97]. However, insufficient details are provided in regard to ROC analysis (e.g., no AUCs confidence intervals are reported) or any utility index [97]. Furthermore, Lei et al. reported the Q^*∗*^ index which reflect the point on the SROC (summary receiver operating characteristic curve) at which the Se is equal with Sp that could be useful in specific clinical situations [97].

The number needed to diagnose (NND) and number needed to misdiagnose (NNM) are currently used in the identification of the cutoff value on continuous diagnostic test results [172, 177], in methodological articles, or teaching materials [161, 178, 179]. The NND and NNM are less frequently reported in the evaluation of the accuracy of a diagnostic test. Several examples identified in the available scientific literature are as follows: color duplex ultrasound in the diagnosis of carotid stenosis [180], culture-based diagnosis of tuberculosis [181], prostate-specific antigen [182, 183], endoscopic ultrasound-guided fine needle biopsy with 19-gauge flexible needle [184], number needed to screen-prostate cancer [185, 186], the integrated positron emission tomography/magnetic resonance imaging (PET/MRI) for segmental detection/localization of prostate cancer [187], serum malondialdehyde in the evaluation of exposure to chromium [188], the performances of the matrix metalloproteinase-7 (MMP-7) in the diagnosis of epithelial injury and of biliary atresia [189], lactate as a diagnostic marker of pleural and abdominal exudate [190], the Gram stain from a joint aspiration in the diagnosis of pediatric septic arthritis [191], and performances of a sepsis algorithm in an emergency department [192]. Unfortunately, the NND or NNM point estimators are not all the time reported with the associated 95% confidence intervals [161, 180, 181, 186, 187, 190, 191].

The reporting of the clinical utility index (CUI) is more frequently seen in the evaluation of a questionnaire. The grades not the values of CUIs were reported by Michell et al. [193] in the assessment of a semistructured diagnostic interview as a diagnostic tool for the major depressive disorder. Johansson et al. [194] reported both the CUI + value and its interpretation in cognitive evaluation using Cognistat. The CUI+/CUI− reported by Michell et al. [195] on the patient health questionnaire for depression in primary care (PHQ-9 and PHQ-2) is reported as a value with associated 95% confidence interval as well as interpretation. The CUI+ and CUI− values and associated confidence intervals were also reported by Fereshtehnejad et al. [196] in the evaluation of the screening questionnaire for Parkinsonism but just for the significant items. Fereshtehnejad et al. [196] also used the values of CUI+ and CUI− to select the optimal screening items whenever the value of point estimator was higher than 0.63. Bartoli et al. [197] represented the values of CUI graphically as column bars (not necessarily correct since the CUI is a single value, and a column could induce that is a range of values) in the evaluation of a questionnaire for alcohol use disorder on different subgroups. The accurate reporting of CUIs as values and associated confidence intervals could also be seen in some articles [198, 199], but is not a common practice [200–207].

Besides the commercial statistical programs able to assist researchers in conducting an accuracy analysis for a diagnostic test, several free online (Table 12) or offline applications exist (CATmaker [208] and CIcalculator [209]).

Smartphone applications have also been developed to assist in daily clinical practice. The *DocNomo* application for iPhone/iPad free application [210] allows calculation of posttest probability using the two-step Fagan nomogram. Other available applications are *Bayes' posttest probability calculator*, *EBM Tools app*, and *EBM Stats Calc*. Allen et al. [211] and Power et al. [212] implemented two online tools for the visual examination of the effect of Se, Sp, and prevalence on TP, FP, FN, and TN values and the evaluation of clinical accuracy and utility of a diagnostic test [213]. Furthermore, they have underconstructed the evaluation of the uncertainties in assessing test accuracy when the reference standard is not perfect as support for the evidence-based practice.

## 4. Cost-Benefit Analysis

The studies conducted in phase III and IV in the investigation of a diagnostic test could be covered under the generic name of cost-benefit analysis. Different aspects of the benefit could be investigated such as societal impact (the impact on the society), cost-effectiveness (affordability), clinical efficacy or effectiveness (effects on the outcome), cost-consequence analysis, cost-utility analysis, sensitivity analysis (probability of disease and/or recurrence, cost of tests, impact on QALY (quality-adjusted life-year), and impact of treatment), and analytical performances (precision, linearity, and cost-effectiveness ratio) [214]. Thus, the evaluation of diagnostic tests benefits could be investigated from different perspectives (e.g., societal, health-care system, and health-care provider) and considering different items (e.g., productivity, patient and family time, medication, and physician time) [215]. Furthermore, an accurate comparison of two diagnostic tests must consider both the accuracy and benefit/harm in the assessment of the clinical utility [216, 217]. Generally, then cost-benefit analysis employs multivariate and multifactorial analysis using different designs of the experiment, including survival analysis, and the statistical approach is selected according to the aim of the study. Analysis of relationships is done using correlation method (Person's correlation (*r*) when the variables (two) are quantitative and normal distributed, and a linear relation is assuming between them; Spearman's (*ρ*) or Kendall's (*τ*) correlation coefficient otherwise; it is recommended to use Kendall's tau instead of Spearman's rho when data have ties [218]) or regression analysis when the nature of the relationship is of interest and an outcome variable exists [219]. The statistical methods applied when cost-benefit analysis is of interest are not discussed in detail here, but the basic requirements in reporting results are as follows [220–225]:

- Correlation analysis: give summary statistic according to the distribution of data (with associated 95% confidence intervals when appropriate, for both baseline data and outcome data), graphical representation as scatter plot, use correct symbol of the correlation coefficient and associate the *P* value along with the sample size, report missing data, and report the check for influential/outliers.
- Multivariate or multifactorial analysis: summary of the check of assumptions (plots, tests, and indicators), provide the plot of the model, give the model with coefficients, standard error of the coefficients and associated *P* values or 95% confidence intervals, determination coefficient of the mode, standard error of the model, statistic and *P* value of the model, provide the sample size, give the number of missing data for each predictor, and adjusted and unadjusted metrics (e.g., OR in logistic regression and HR (hazard ratio) in survival analysis).

Miglioretti et al. [98] investigated the link between radiation exposure of children through the CT examination and the risk of cancer. They reported a trend of the use in the CT which increased from 1996 to 2005, a plateau between 2005 and 2007 followed by a decrease till 2010. The number of CT scans was reported per 1,000 children. Regardless of the anatomical CT scan, the average effective doses were expressed as mean and percentiles (25^th^, 50^th^, 75^th^, and 95^th^), while the dose exceeding 20 mSv was reported as percentages. The mean organ dose was also reported and the lifetime attributable risk of solid cancer or leukemia, as well as some CT scans leading to one case of cancer per 10,000 scans [98]. The reported numbers and risks were not accompanied by the 95% confidence intervals [98] excepting the estimated value of the total number of future radiation-induced cancers related to pediatric CT use (they named it as uncertainty limit).

Dinh et al. [99] evaluated the effectiveness of a combined screening test (fecal immunological test and colonoscopy) for colorectal cancer using the Archimedes model (human physiology, diseases, interventions, and health-care systems [226]). The reported results, besides frequently used descriptive metrics, are the health utility score [227], cost per person, quality-adjusted life-years (QALYs) gained per person, and cost/QALYs gain as numerical point estimators not accompanied by the 95% confidence interval.

Westwood et al. [228] conducted a secondary study to evaluate the performances of the high-sensitivity cardiac troponin (hs-cTn) assays in ruling-out the patients with acute myocardial infarction (AMI). Clinical effectiveness using metrics such as Se, Sp, NLR, and PLR (for both any threshold and 99^th^ percentile threshold) was reported with associated 95% confidence intervals. As the cost-effectiveness metrics the long-term costs, cost per life-year (LY) gained, quality-adjusted life-years (QALYs), and costs/QALYs were reported with associated 95% confidence intervals for different Tn testing methods. Furthermore, the incremental cost-effectiveness ratio (ICER) was used to compare the mean costs of two Tn testing methods along with the multivariate analysis (reported as estimates, standard error of the estimate, and the distribution of data).

Tiernan et al. [100] reported the changes in the clinical practice for the diagnosis of latent tuberculosis infection (LTBI) with interferon-gamma release assay, namely, QuantiFERON-TB Gold In-Tube (QFT, Cellestis, Australia). Unfortunately, the reported outcome was limited to the number of changes in practice due to QFT as absolute frequency and percentages [100].

## 5. Limitations and Perspectives

The current paper did not present either detail regarding the research methodology for diagnostic studies nor the critical appraisal of a paper presenting the performances of a diagnostic test because these are beyond the aim. Extensive scientific literature exists regarding both the design of experiments for diagnostic studies [4, 15, 92, 229, 230] and the critical evaluation of a diagnostic paper [231–234]. As a consequence, neither the effect of the sample size on the accuracy parameters, or the *a priori* computation of the sample size needed to reach the level of significance for a specific research question, nor the *a posteriori* calculation of the power of the diagnostic test is discussed. The scientific literature presenting the sample size calculation for diagnostic studies is presented in the scientific literature [235–238], but these approaches must be used with caution because the calculations are sensitive and the input data from one population are not a reliable solution for another population, so the input data for sample size calculation are recommended to come from a pilot study. This paper does not treat how to select a diagnostic test in clinical practice, the topic being treated by the evidence-based medicine and clinical decision [239–241].

Health-care practice is a dynamic field and records rapid changes due to changes in the evolution of known diseases, the apparition of new pathologies, the life expectancy of the population, progress in information theory, communication and computer sciences, development of new materials, and approaches as solutions for medical problems. The concept of personalized medicine changes the way of health care, the patient becomes the core of the decisional process, and the applied diagnostic methods and/or treatment closely fit the needs and particularities of the patient [242]. Different diagnostic or monitoring devices such as wearable health monitoring systems [243, 244], liquid biopsy or associated approaches [245, 246], wireless ultrasound transducer [247], or other point-of-care testing (POCT) methods [248, 249] are introduced and need proper analysis and validation. Furthermore, the availability of big data opens a new pathway in analyzing medical data, and artificial intelligence approaches will probably change the way of imaging diagnostic and monitoring [250, 251]. The ethical aspects must be considered [252, 253] along with valid and reliable methods for the assessment of old and new diagnostic approaches that are required. Space for methodological improvements exists, from designing the experiments to analyzing of the experimental data for both observational and interventional approaches.

## 6. Concluding Remarks

Any diagnostic test falls between perfect and useless test, and no diagnostic test can tell us with certainty if a patient has or not a particular disease. No ideal diagnostic tests exist, so any test has false-positive and false-negative results.

The metric reported in the assessment of the precision (variability analysis) or accuracy of a diagnostic test must be presented as point indicators and associated 95% confidence interval, and the thresholds for interpretation are applied to the confidence intervals.

The correct evaluation of performances of two methods measuring the same outcome is done with the Bland and Altman plot (evaluate the bias of the difference between two methods) not correlation or agreement (assess the association between two measurements) analysis.

A *gold standard* test is mandatory in the evaluation of the accuracy of a test. Both sensitivity and specificity with 95% confidence intervals are reported together to allow a proper interpretation of the accuracy. Based on these values, the clinical utility index is used to support the rule-in and/or rule-out and thus respectively the usefulness of a diagnostic test as identification of the disease or in screening.

The correct interpretation of positive and negative predictive values is just made if the prevalence of the disease is known.

The sensitivity and specificity must be reported any time when Youden's index is given. Report the ROC analysis by providing AUC with associated 95% confidence interval, the threshold according to Youden's index, sensitivity, and specificity with 95% confidence intervals.

Report full descriptive and inferential statistics associated with the benefits analysis. Multivariate or multifactorial analysis could be used to test the cost-benefit of a diagnostic test, and the good practice in reporting such analysis must be strictly followed by providing the full model with the values of coefficients associated to the predictors and measures of variability, significance of both models and each coefficient, and risk metrics with associated 95% confidence intervals when appropriate (e.g., relative risk and hazard ratio).

## Figures and Tables

**Table 1:** Anatomy on phases of a diagnostic test.

Phase	What?	Design
I	Determination of normal ranges (pharmacokinetics, pharmacodynamics, and safe doses)	Observational studies on healthy subjects

II	Evaluation of diagnosis accuracy	Case-control studies on healthy subjects and subjects with the known (by a *gold standard* test) and suspected disease of interest (i) Phase IIa: healthy subjects and subjects with the known disease of interest, all diagnosed by a *gold standard* method (ii) Phase IIb: testing the relevance of the disease severity (evaluate how a test works in ideal conditions) (iii) Phase IIc: assess the predictive values among subjects with suspected disease

III	Evaluation of clinical consequences (benefic and harmful effects) of introducing a diagnostic test	Randomized control trials, randomization determine whether a subject receive or not the diagnosis test

IV	Determination of the long-term consequences of introducing a new diagnostic test into clinical practice	Cohort studies of consecutive participants to evaluate if the diagnostic accuracy of a test in practice corresponds to predictions from systematic reviews of phase III trials

Adapted from [7].

**Table 2:** Anatomy of the role of a diagnostic test.

Role	What?	Example (ref.)
Confirmation/exclusion	Confirm (rule-in) or exclude (rule-out) the disease	Brain natriuretic peptide: diagnostic for left ventricular dysfunction [33]
Triage	An initial test that could be rapidly applied and have a small number of false-positive results	Renal Doppler resistive index: hemorrhagic shock in polytrauma patients [34]
Monitoring	A repeated test that allows assessing the efficacy of an intervention	Glycohemoglobin (A1c Hb): overall glycemic control of patients with diabetes [35]
Prognosis	Assessment of an outcome or the disease progression	PET/CT scan in the identification of distant metastasis in cervical and endometrial cancer [36]
Screening	Presence of the disease in apparently asymptomatic persons	Cytology test: screening of cervical uterine cancer [37]

**Table 3:** Diagnosis test result: type of data.

Data	Example (ref.)
Qualitative dichotomial	Positive/negative or abnormal/normal (i) Endovaginal ultrasound in the diagnosis of normal intrauterine pregnancy [39] (ii) QuantiFERON-TB test for the determination of tubercular infection [40]

Qualitative ordinal	(i) Prostate bed after radiation therapy: definitely normal/probably normal/uncertain/probably abnormal/definitely abnormal [41] (ii) Scores: Apgar score (assessment of infants after delivery): 0 (no activity, pulse absent, floppy grimace, skin blue or pale, and respiration is absent) to 10 (active baby, pulse over 100 bps, prompt response to stimulation, pink skin, and vigorous cry) [42]; Glasgow coma score: eye opening (from 1 = no eye opening to 4 = spontaneously), verbal response (from 1 = none to 5 = patient oriented), and motor response (from 1 = none to 6 = obeys commands) [43]; Alvarado score (the risk of appendicitis) evaluates 6 clinical items and 2 laboratory measurements and had an overall score from 0 (no appendicitis) to 10 (“very probable” appendicitis) [44]; and sonoelastographic scoring systems in evaluation of lymph nodes [45] (iii) Scales: quality-of-life scales (SF-36 [46], EQ-5D [47, 48], VascuQoL [49, 50], and CIVIQ [51]) and pain scale (e.g., 0 (no pain) to 10 (the worst pain)) [52]

Qualitative nominal	(i) Apolipoprotein E gene (ApoE) genotypes: E2/E2, E2/E3, E2/E4, E3/E3, E3/E4, and E4/E4 [53, 54] (ii) SNP (single-nucleotide polymorphism) of IL-6: at position −174 (rs1800795), −572 (rs1800796), −596 (rs1800797), and T15 A (rs13306435) [55]

Quantitative discrete	(i) Number of bacteria in urine or other fluids [56] (ii) Number of contaminated products with different bacteria [57] (iii) Glasgow aneurysm score (= age in years + 17 for shock + 7 for myocardial disease + 10 for cerebrovascular disease + 14 for renal disease) [58]

Quantitative continuous	(i) Biomarkers: chitotriosidase [59], neopterin [60], urinary cotinine [61], and urinary cadmium levels [61] (ii) Measurements: resistivity index [62], ultrasound thickness [63], and interventricular septal thickness [64]

**Table 4:** Statistical methods in the assessment of the utility of a diagnostic test.

Phase	Clinical question	Objective(s)	Statistics for results	Example (ref.)
I	Which are the normal ranges of values of a diagnostic test?	Determination of the range of values on healthy subjects	Centrality and dispersion (descriptive) metrics: (i) mean (SD), where SD = standard deviation, if data follow the normal distribution; (ii) otherwise, median (*Q*1 − *Q*3), where *Q*1 = 25^th^ percentile and *Q*3 = 75^th^ percentiles	(i) Levels of hepcidin and prohepcidin in healthy subjects [93] (ii) plasma pro-gastrin-releasing peptide (ProGRP) levels in healthy adults [94]

I	Is the test reproducible?	Variability: (i) Intra- and interobserver (ii) Intra- and interlaboratory	(i) Agreement analysis: % (95% confidence interval) and agreement coefficients (dichotomial data: Cohen, ordinal data: weighted kappa, numerical: Lin's concordance correlation coefficient, and Bland and Altman diagram) (ii) Variability analysis: Coefficient of variation, distribution of differences	(i) Intra- and interobserver variability of uterine measurements [95] (ii) Interlaboratory variability of cervical cytopathology [96] (iii) Concordance between tuberculin skin test and QuantiFERON in children [40]

II	Is the test accurate? Which are performances of the diagnostic test?	Determine the accuracy as compared to a gold standard test	(i) Metrics (dichotomial outcome): Se (sensitivity), Sp (specificity), PPV (predictive positive value), NPV (negative predictive value), and DOR (diagnostic odds ratio) (ii) Clinical performances (dichotomial outcome): PLR (positive likelihood ratio) and NLR (negative likelihood ratio) (iii) Threshold identification (numerical or ordinal with a minimum of five classes outcome): ROC (receiver operating characteristic curve) analysis	(i) Digital breast tomosynthesis for benign and malignant lesions in breasts [97] (ii) Chitotriosidase as a marker of inflammatory status in critical limb ischemia [59] (iii) Sonoelastographic scores to discriminate between benign and malignant cervical lymph nodes [45]

III	Which are the costs, risk, and acceptability of a diagnostic test?	(i) Evaluation of beneficial and harmful effects (ii) Cost-effective analysis	Retrospective or prospective studies: (i) beneficial (e.g., improvement of clinical outcome) or harmful effects (e.g., morbidity and mortality) by proportions, risk ratio, odds ratio, hazard ratio, the number needed to treat, and rates and ratios of desirable or undesirable outcomes (ii) cost-effective analysis (mean cost and quality-adjusted life years (QALYs))	(i) The computed tomography in children, the associated radiation exposure, and the risk of cancer [98] (ii) Healthcare benefit and cost-effectiveness of a screening strategy for colorectal cancer [99]

IV	Which are the consequences of introducing a new diagnostic test into clinical practice?	(i) Does the test result affect the clinical decision?	(i) Studies of pre- and posttest clinical decision-making (ii) %: abnormal, of discrepant results, of tests leading to change the clinical decisions (iii) Costs: per abnormal result, decision change	(i) Does the interferon-gamma release assays (IGRAs) change the clinical management of patients with latent tuberculosis infection (LTBI)? [100]

**Table 5:** Online resources for confidence intervals calculation: coefficient of variation.

What?	URL (accessed on August 26, 2018)
Two-sided confidence interval (CI) for s CV^a^	https://www1.fpl.fs.fed.us/covnorm.dcd.html https://community.jmp.com/kvoqx44227/attachments/kvoqx44227/scripts/77/1/CI%20for%20CV%202.jsl
One-sided CI^a^ Lower bound Upper bound	https://www1.fpl.fs.fed.us/covlow.html https://www1.fpl.fs.fed.us/covup.html
Two-sided CI for s CV^b^	https://www1.fpl.fs.fed.us/covln.html
Ratio of two CVs^a^	https://www1.fpl.fs.fed.us/covratio.html

^a^Normal distribution and ^b^lognormal distribution.

**Table 6:** Intra- and interclass correlation coefficients and concordance correlation coefficient: an empirical assessment of the strength of agreement.

Agreement	Continuous measurement	Ultrasound fetal measurements	Semiautomated measurements
Very good	*ρ*_c_ > 0.99	*ρ*_c_ > 0.998	*ρ*_c_ > 0.90
Good	0.95 < *ρ*_c_ ≤ 0.99	0.99 < *ρ*_c_ ≤ 0.998	0.80 < *ρ*_c_ ≤ 0.90
Moderate	0.90 < *ρ*_c_ ≤ 0.95	0.98 < *ρ*_c_ ≤ 0.99	0.65*ρ*_c_ ≤ 0.80
Poor	0.70 < *ρ*_c_ ≤ 0.90	0.95 < *ρ*_c_ ≤ 0.98	*ρ*_c_ < 0.65
Very poor	*ρ*_c_ < 0.70	*ρ*_c_ < 0.95	

Source [141, 142].

**Table 7:** 2 × 2 contingency generic table.

Diagnostic test result	Disease present	Disease absent	Total
Positive	TP (true positive)	FP (false positive)	TP + FP
Negative	FN (false negative)	TN (true negative)	FN + TN
Total	TP + FN	FP + TN	*n* = TP + FP + FN + TN

Total on the rows represents the number of subjects with positive and respectively negative test results; total on the columns represents the number of subjects with (disease present) and respectively without (disease absent) the disease of interest; and the classification as test positive/test negative is done using the cutoff value for ordinal and continuous data.

**Table 8:** Standard statistic indicators used to evaluate diagnostic accuracy.

Statistic (Abb)	Formula	Remarks
Sensitivity (Se)	TP/(TP + FN)	(i) The highest the Se, the smallest the number of false negative results (ii) High Se: (a) a negative result rules-out (SnNOUT) (b) suitable for screening (ruling-out)

Specificity (Sp)	TN/(TN + FP)	(i) The highest the Se, the smallest the number of false-positive results (ii) High Sp: (a) a positive result rules-in (SpPIN) (b) It is suitable for diagnosis (ruling-in)

Accuracy index (AI)	(TP + TN)/(TP + FP + FN + TN)	(i) Give information regarding the cases with the right diagnosis (ii) It is difficult to convert its value to a tangible clinical concept (iii) It is affected by the prevalence of the disease

Youden's index (*J*) [159]	Se + Sp − 1	(i) Sums the cases wrongly classified by the diagnostic test (ii) Assess the overall performance of the test. *J* = 0, if the proportion of positive tests is the same in the group with/without the disease. *J* = 1, if no FPs or FNs exist (iii) Misleading interpretation in comparison of the effectiveness of two tests (iv) Used to identify the best cutoff on ROC analysis: its maximum value corresponds to the highest distance from diagonal

Positive predictive value (PPV)^*∗*^	TP/(TP + FP)	(i) Answer the question “what is the chance that a person with a positive test truly has the disease?” (ii) Clinical applicability for a particular subject with a positive test result (iii) It is affected by the prevalence of the disease

Negative predictive value (NPV)^*∗*^	TN/(TN + FN)	(i) Answer the question “what is the chance that a person with a negative test truly not to have the disease?” (ii) Clinical applicability for a particular subject with a negative test result (iii) It is affected by the prevalence of the disease

Positive likelihood ratio (PLR/LR+)^*∗*^	Se/(1 − Sp)	(i) Indicates how much the odds of the disease increase when a test is positive (indicator to rule-in) (ii) PLR (the higher, the better) (a) > 10 ⟶ convincing diagnostic evidence (b) 5 < PLR < 10 ⟶ strong diagnostic evidence

Negative likelihood ratio (NLR/LR−)^*∗*^	(1 − Se)/Sp	(i) Indicates how much the odds of the disease decrease when a test is negative (indicator to rule-out) (ii) NLR (the lower, the better) (a) < 0.1 ⟶ convincing diagnostic evidence (b) 0.2 < PLR < 0.1 ⟶ strong diagnostic evidence
Diagnostic odds ratio (DOR)^*∗∗*^ [160]	(TP/FN)/(FP/TN) [Se/(1 − Se)]/[(1 − Sp)/Sp] [PPV/(1 − PPV)]/[(1 − NPV)/NPV] PLR/NLR	(i) High DOR indicates a better diagnostic test performance (ranges from 0 to infinite). A value of 1 indicates a test not able to discriminate between those with and those without the disease (ii) Combines the strengths of Se and Sp (iii) Useful to compare different diagnostic tests (iv) Not so useful when the aim is to *rules-in* or *rules*-*out* (v) Convenient indicator in the meta-analysis

Posttest odds (PTO)^*∗*^ Posttest probability (PTP)^*∗*^	Pretest odds (prevalence/(1 − prevalence)) × LR PTO/(PTO + 1)	(i) Gives the odds that the patient has to the target disorder after the test is carried out (ii) Gives the proportion of patients with that particular test result who have the target disorder

All indicators excepting *J* are reported with associated 95% confidence intervals; ROC = receiver-operating characteristic; ^*∗*^patient-centered indicator; TP = true positive; FP = false positive; FN = false negative; TN = true negative; and PPV and NPV depend on the prevalence (to be used only if (no. of subjects with disease)/(no. of patients without disease) is equivalent with the prevalence of the disease in the studied population).

**Table 9:** Other metrics used to evaluate diagnosis accuracy.

Statistic (Abb)	Formula	Remarks
Number needed to diagnose (NND) [161]	1/[Se − (1 − Sp)]1/*J*	(i) The number of patients that need to be tested to give one correct positive test result (ii) Used to compare the costs of different tests

Number needed to misdiagnose (NNM) [162]	1/[1 − (TP + TN)/*n*]	(i) The highest the NNM, the better the diagnostic test

Clinical utility index (CUI) [163, 164]	CUI+ = Se × PPV CUI− = Sp × NPV	(i) Gives the degree to which a diagnostic test is useful in clinical practice (ii) Interpretation: CUI > 0.81 ⟶ *excellent utility*; 0.64 ≤ CUI < 0.81 ⟶ *good utility*; 0.49 ≤ CUI < 0.64 ⟶ *fair utility*; 0.36 ≤ CUI < 0.49 ⟶ *poor utility*; and CUI < 0.36 ⟶ *very poor utility*

Abb = abbreviation; all indicators excepting *J* are reported with associated 95% confidence intervals; TP = true positive; FP = false positive; FN = false negative; and TN = true negative.

**Table 10:** Metrics for global test accuracy evaluation or comparisons of performances of two tests.

Statistic (Abb)	Method	Remarks
Area under the ROC curve (AUC)	(i) Nonparametric (no assumptions): empirical method (estimated AUC is biased if only a few points are in the curve) and smoothed-curve methods such as kernel density method (not reliable near the extremes of the ROC curve) (ii) Parametric (the distributions of the cases and controls are normal): binomial method (tighter asymptotic confidence bounds for samples less than 100)	(i) AUC = 1 ⟶ perfect diagnostic test (perfect accuracy) (ii) AUC ∼ 0.5 ⟶ random classification (iii) 0.9 < AUC ≤ 1 ⟶ excellent accuracy classification (iv) 0.8 < AUC ≤ 0.9 ⟶ good accuracy (v) 0.7 < AUC ≤ 0.8 ⟶ worthless

Partial area under the curve (pAUC)	(i) Nonparametric (no assumptions) (ii) Parametric: using the binomial assumption	(i) Looks to a portion AUC for a predefined range of interest (ii) Depends on the scale of possible values on the range of interest (iii) Has less statistical precision compared to AUC

Diagnostic odds ratio (DOR)	(i) Must use the same fixed cutoff (ii) Most useful in a meta-analysis when two or more tests are compared	(i) DOR = 1 ⟶ test (ii) DOR increases as ROC is closer to the top left-hand corner of the ROC plot (iii) The same DOR could be obtained for different combinations of Se and Sp

TP fraction for a given FP fraction (TPF_FPF_)	(i) Need the same false-positive fraction	(i) Useful to compare two different tests at a specific FPF (decided based on clinical reasoning), especially when the ROC curves cross

Comparison of two tests	(i) Comparison of AUC of two different tests (ii) Absolute difference (Se_A_ − Se_B_) or ratio (Se_A_/Se_B_), where A is one diagnostic test and B is another diagnostic test	(i) Apply the proper statistical test; each AUC must be done relative to the “gold-standard” test (ii) Test A better than B if absolute difference is > 0; ratio > 1

Abb = abbreviation; all indicators are reported with associated 95% confidence intervals; ^*∗*^patient-centered indicator; TP = true positive; FP = false positive; FN = false negative; and TN = true negative.

**Table 11:** Performances metrics for body mass index (BMI) as an anthropometric marker for breast cancer.

Indicator	Cutoff–BMI (kg/m^2^)						
	19.5	22.5	25.5	29.5	32.5	35.5	38.5
TP (true positives)	100	96	87	67	43	25	13
FP (false positives)	200	176	117	24	3	1	0
TN (true negatives) off	0	24	83	176	197	199	200
FN (false negatives)	0	4	13	33	57	75	87
Se (sensitivity)	1	1	0.87	0.67	0.43	0.25	0.13
Sp (specificity)	0	0.10	0.42	0.88	0.99	0.99	1
PPV (positive predictive value)	0.33	0.40	0.43	0.74	0.94	0.96	1
NPV (negative predictive value)	n.a.	0.90	0.87	0.84	0.78	0.73	0.70
PLR (positive likelihood ratio)	1.00	1.10	1.49	5.58	28.7	50.0	n.a.
NLR (negative likelihood ratio)	n.a.	0.30	0.31	0.38	0.58	0.75	0.84
AI (accuracy index)	0.33	0.40	0.57	0.81	0.80	0.75	0.71
CUI+ (clinical utility index positive)	0.33	0.30	0.37	0.47	0.40	0.24	0.13
CUI− (clinical utility index negative)	n.a.	10	0.36	0.74	0.76	0.72	0.70

**Table 12:** Online applications for diagnostic tests: characteristics.

Name	Input	Output
Diagnostic test calculator^a^	TP, FP, TN, FN *OR* Prevalence AND Se AND Sp AND sample size *OR* Prevalence AND PLR AND NLR AND sample size	Prevalence AND Se AND Sp AND PLR AND NLR Fagan diagram

Diagnostic test calculator evidence-based medicine toolkit^b^	TP, FP, TN, FN	Se, Sp, PPV, NPV, PLR, NLR with associated 95% confidence intervals Posttest probability graph

MedCalc: Bayesian analysis model^c^	Prevalence AND Se AND Sp *OR* TP, FP, TN, FN	PPV, NPV, LPR, NLR, posttest probability

MedCalc^d^	TP, FP, TN, FN	Se, Sp, PPV, NPV, PLR, NLR, prevalence, AI with associated 95% confidence intervals

Clinical calculator 1^e^	TP, FP, TN, FN	Se, Sp, PPV, NPV, PLR, NLR, prevalence, AI with associated 95% confidence intervals

Clinical utility index calculator^f^	TP, TN, total number of cases, the total number of noncases	Se, Sp, PPV, NPV, PLR, NLR, prevalence, AI with associated 95% confidence intervals

DiagnosticTest^g^	Number of positive and negative gold standard results for each level of the new diagnostic test	Se, Sp, PPV, NPV, PLR, NLR, AI, DOR, Cohen's kappa, entropy reduction, and a bias Index ROC curve if > 2 levels for all possible cutoff

Simple ROC curve analysis^h^	Absolute frequencies for false positive and the true positive for up to ten diagnostic levels	Cumulative rates (false positive and true positive) and ROC curve (equation, *R*^2^, and AUC)

ROC analysis^i^	Five different type of input data: an example for each type is provided	Se, Sp, AI, positive cases missed, negative cases missed, AUC, ROC curve

AUSVET: EpiTools^j^	TP, FP, TN, FN	Different tools from basic accuracy to comparison of two diagnostic tests to ROC analysis

All URLs were retrieved on April 20, 2019. TP = true positive; FP = false positive; FN = false negative; TN = true negative; Se = sensitivity; Sp = specificity; AI = accuracy index; PPV = positive predictive value; NPV = negative predictive value; PLR = positive likelihood ratio; NLR = negative likelihood ratio; DOR = diagnostic odds ratio; ROC = receiver operating characteristic; AUC = area under the ROC curve; ^a^http://araw.mede.uic.edu/cgi-bin/testcalc.pl; ^b^https://ebm-tools.knowledgetranslation.net/calculator/diagnostic/; ^c^http://www.medcalc.com/bayes.html; ^d^https://www.medcalc.org/calc/diagnostic_test.php; ^e^http://vassarstats.net/clin1.html; ^f^http://www.psycho-oncology.info/cui.html; ^g^http://www.openepi.com/DiagnosticTest/DiagnosticTest.htm; ^h^http://vassarstats.net/roc1.html; ^i^http://www.rad.jhmi.edu/jeng/javarad/roc/JROCFITi.html; and ^j^http://epitools.ausvet.com.au/content.php?page=TestsHome.
